# Dynamics of *Marenzelleria* spp. Biomass and Environmental Variability: A Case Study in the Neva Estuary (The Easternmost Baltic Sea)

**DOI:** 10.3390/biology13120974

**Published:** 2024-11-26

**Authors:** Sergey M. Golubkov, Mikhail S. Golubkov

**Affiliations:** Zoological Institute of Russian Academy of Sciences, Universitetskaya Emb. 1, 199034 Saint-Petersburg, Russia; golubkov_ms@mail.ru

**Keywords:** biological invasions, invasion ecology, invasive species, alien species, “boom and bust” pattern, invertebrates, coastal ecosystems, community structure, habitat selection, environmental filter

## Abstract

Predicting which non-indigenous species (NISs) will establish persistent invasive populations and cause significant ecosystem changes remains an important environmental challenge. We analyzed the spatial and temporal dynamics of the entire zoobenthos and the biomass of *Marenzelleria* spp., one of the most successful invaders in the Baltic Sea, in the Neva estuary in 2014–2023. A considerable decrease in *Marenzelleria* biomass was observed in the second half of the study period, which was accompanied by a sharp increase in the dominance of opportunistic oligochaete and chironomid species. Our one-way analysis of variance showed that communities with high *Marenzelleria* biomass had significantly higher diversity and biomass of native benthic crustaceans compared to communities with low alien polychaetes biomass. A high biomass of *Marenzelleria* was observed in biotopes characterized by low temperatures, high salinity, low plankton primary production and chlorophyll concentration. The results of PCA and one-way ANOVA indicated that these factors significantly influenced the spatial and temporal dynamics of the polychaete biomass. More detailed studies of the responses of NISs to environmental variables are needed to better understand and anticipate their dynamics in different regions of the Baltic Sea in relation to climate warming and anthropogenic impacts.

## Simple Summary

Identifying the factors that drive the dynamics of invasive species is critical for predicting potential changes in recipient ecosystems and for implementing effective legislation on non-native species. The polychaetes *Marenzelleria* spp. are among the most successful alien species that have recently invaded European seas. Using ten years of observations on environmental variables and the distribution of biomass of this and other benthic invertebrates in the Neva estuary, we determined the main factors influencing the abundance and dynamics of this invasive species. Statistical analysis revealed that communities dominated by alien polychaetes were positively associated with water salinity and biotope depth while showing negative associations with water temperature, plankton primary production and chlorophyll concentration. Fluctuations in these variables, largely driven by climate change, may have contributed to the recent decline in the population of this invasive species in the estuary.

## 1. Introduction

Biological invasions have significant impacts on Earth’s ecosystems, necessitating a deeper understanding of the factors that influence these phenomena [1]. There is an urgent need to establish a dedicated framework that provides practical tools for environmental managers tasked with implementing non-indigenous species (NIS) legislation [2,3]. NISs can alter species composition and processes within ecosystems through various mechanisms and to differing extents [4]. In some instances, these species act as ecosystem engineers, radically transforming the structure and function of their new environments [5]. Therefore, predicting which NISs will establish persistent invasive populations and induce substantial ecosystem changes remains a critical environmental challenge [6]. Long-term studies of NISs that encompass a wide range of environmental factors and ecosystem responses are essential for understanding the alterations they impose on communities and ecosystems [7].

The polychaetes *Marenzelleria* spp. are among the most successful benthic invaders in the northern seas of Europe [8,9]. They have also been recently documented in the Azov, Black and Caspian seas [9]. Their introduction into the Baltic Sea was facilitated by the low biodiversity of the local benthic fauna and high levels of eutrophication in many areas [10]. These NISs have emerged as ecosystem engineers in numerous locations [5]. Their ability to create deep burrows and aerate bottom sediments significantly affects the exchange of nutrients between sediments and the water column [11,12]. Additionally, their capacity for bioturbation is an important trait that facilitates their successful establishment in habitats where local zoobenthic species exhibit limited bioturbation abilities [13]. At high biomass levels, these alien polychaetes can substantially alter food webs in recipient communities as their deep burrows make them less accessible to fish and invertebrate predators [14].

When introduced, a non-native species must pass through environmental and biotic filters [15]. Environmental filters encompass the abiotic conditions that enable species to survive successfully, while biotic filters include resource availability, competition with native species, and predation pressure. There is ongoing debate regarding which type of filter is more critical for successful invasion. Numerous studies have indicated that favorable abiotic environmental factors are paramount for the survival of NISs [16,17,18,19], while others have highlighted the importance of biotic interactions with native species (e.g., [14,20,21]) or a combination of both abiotic and biotic factors [22,23]. This complexity is further compounded by the observation that many alien species exhibit population decline following an initial period of exceptionally high abundance [24]. Regarding the environmental filter influencing *Marenzelleria* populations, a regression tree analysis using a binary recursive partitioning of environmental factors has shown that depth, temperature and salinity are significant variables affecting *Marenzelleria* abundance in the Baltic Sea [10,23]. The lower abundance and occurrence of *Marenzelleria* spp. in coastal regions of the Baltic Sea, particularly near river mouths, may result from low salinity levels, which could hinder worm reproduction [25,26,27]. Nevertheless, during their larval stage, these polychaetes can be dispersed by currents into areas with salinities well below its reproductive thresholds [10,25]. Their populations in such habitats may be sustained by the migration of benthic stages from higher salinity areas [26].

Information regarding the biotic filter affecting *Marenzelleria* populations is relatively scarce. In the Baltic Sea, these NISs are known to rapidly colonize habitats where the density of native zoobenthos species has significantly declined due to hypoxia [10,28]. The low species richness of indigenous zoobenthic organisms has also contributed to the dominance of alien worms following their invasion [10]. It has been suggested that this species complex competes with native Baltic species, such as the mollusk *Macoma balthica* (Linnaeus) and the amphipod *Monoporeia affinis* (Lindström) [29,30]. However, only adult *M. balthica* has been shown to exert significant negative effects on *Marenzelleria* [29]. Additionally, negative relationships between the biomass of alien polychaetes and native oligochaetes have been observed in the eastern part of the Gulf of Finland [14]. In contrast, invertebrate predators and fish do not appear to significant impact these polychaetes, as they can effectively hide in the deep burrows they dug [14].

In the Neva estuary, located in the easternmost part of the Gulf of Finland, *Marenzelleria* spp. were first documented in 1997; however, their populations only experienced explosive growth beginning in 2009. This surge followed several events of benthic hypoxia, which led to a substantial decline in the biomass of native zoobenthic communities [28]. Recently, two alien species of the genus *Marenzelleria* have been identified in the Gulf of Finland [9,31]. One of these, *M. neglecta* Sikorski and Bick, invaded from the North Atlantic coast of the United States and predominantly inhabits shallow, well-warmed areas of the gulf. The other species, *M. arctia* (Chamberlin), originated from the coastal Arctic regions of Eurasia and is found in deeper habitats below the thermocline. The high biomass of these alien polychaetes persisted until the mid-2010s, after which it began to decline rapidly [14]. The reasons for this decline remain poorly understood and may be linked to climate fluctuations that affect the ecosystem and biological communities of the Neva estuary [32,33] as well as biotic interactions within zoobenthic communities. To elucidate factors contributing to spatial and temporal variability in alien polychaete populations, we examined the relationship between their biomass and environmental variables, including zoobenthic community composition.

## 2. Materials and Methods

### 2.1. Study Site and Sampling

The Neva Estuary, the largest estuary of the Baltic Sea, is located in the easternmost part of the Gulf of Finland. It is brackish, non-tidal, and shallow in its upper part with horizontal and vertical salinity gradients. Its morphometric and hydrochemical features have previously been described in detail in several publications (e.g., [34,35,36]). The largest metropolis in the Baltic region, St. Petersburg, with a population of more than 5 million people, is located on the coast of the eastern part of the Neva estuary. Eutrophication, organic pollution and port construction significantly impact the biological communities of the estuary [37,38], and these effects are further exacerbated by climate change [32,33]. The destruction of natural habitats and increased maritime traffic have facilitated the invasion of numerous NISs of macroinvertebrates and fish (e.g., [19,28,39].

**Figure 1:**
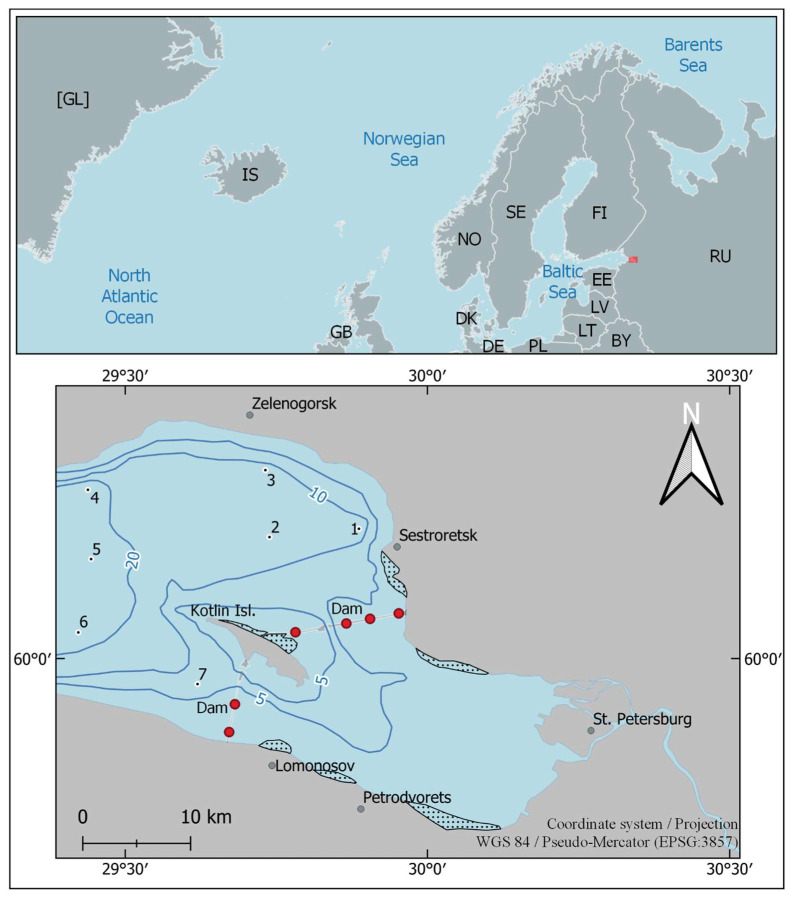
The middle part of the Neva estuary with indication of sampling stations (1–7). Blue lines: isobaths of 5, 10, and 20 m. Black dots in light circles indicate the location of sampling stations. Areas with dots indicate dense reeds. Dam is the St. Petersburg Flood Protection Facility. Red circles—waters gates in the Dam. Red rectangle in the top block of the map shows the location of the Neva estuary. Two-letter country codes are given according to ISO 3166-1 alpha-2 [40].

The study was carried out within the framework of the long-term research conducted by the Zoological Institute RAS in the Neva estuary [19,32,36]. Zoobenthos samples were regularly collected in triplicate at seven stations in the central part of the Neva estuary (Figure 1) using a Van Veen grab (20 × 20 cm) in early August from 2014 to 2023. Alien polychaetes were found at all sampling stations during the study period. The samples were sieved through a 0.25 mm mesh using filtered water and preserved in 4% formaldehyde. The depths at different sampling stations ranged from 7 m at station 7 to 24.5 m at station 5. In the laboratory, invertebrates were removed from samples under a stereomicroscope, identified, counted, and weighed to the nearest 0.1 mg. Animal biomass (wet weight, including mollusk shells) was calculated as the arithmetic mean of three replicates ± standard error of the mean (SEM) and recalculated per square meter of bottom area.

Environmental variables such as salinity (Sal), temperature (Temp), pH, as well as oxidation/reduction potential (Eh) were measured using a CTD90M probe from Sea & Sun Tech (Trappenkamp, Germany). Chlorophyll *a* (CHL) concentration and water turbidity (Turb) were determined using Cyclop-7 sensors connected to a submersible C-6 multi-sensor platform (Turner Designs, San Jose, CA, USA). Water samples to determine the total amount of suspended particles in the water layer above (SMe) and below (SMg) thermocline were taken with a 2 L bathometer. Samples from the upper layer were collected from the surface, the thermocline, and three equally spaced depths, which were mixed to obtain composite samples (10 L). For the lower layer, samples were collected from the thermocline layer, as well as from a depth of 0.5 m above the bottom and from three equally spaced depths, after which they were also mixed to form composite samples. Half a liter of water was collected from the composite samples for the laboratory determination of SM using the gravimetric method via filtration through pre-combusted and pre-weighted Whatman GF/F (0.85 µm pore size) filters [41]. Primary plankton production (PP) and the mineralization of organic matter (MN) in the water column were measured by the light and dark bottle oxygen method [42,43]. A detailed description of the methodologies and experimental design can be found in an earlier article [36].

### 2.2. Statistical Analysis

The dataset was categorized into two groups: Community 1, where the biomass of *Marenzelleria* spp. exceeded the mean for the dataset (>3.84 g m^−2^), and Community 2, where the biomass was below the mean (<3.84 g m^−2^). Using Excel, we calculated the average benthic biomass and standard error of the mean for both communities and determined the proportion of each benthic group in the total biomass.

To explore relationships and patterns in benthic biomass and environmental data, several statistical analyses were performed. Prior to the analyses, the raw data were log-transformed (log_10_) with an added constant of 2 to approximate a normal distribution.

Statistical analysis was performed using R software (version 4.4.0) [44]. To assess the differences between communities, we utilized the Bray–Curtis index, which was calculated using the “vegdist” function from the «vegan» package in R [45]. Hierarchical Clustering Analysis based on the Bray–Curtis dissimilarity index was performed to explore the structural differences between communities. Hierarchical clustering was performed using the “hclust” function from the vegan package in R [45]. Ward’s method (Ward.D2) was used for clustering, aiming to minimize within-cluster variance while maximizing between-cluster variance. The resulting dendrogram illustrates sample groupings based on similarity with clusters defined by a threshold indicated by a rectangle drawn using the “rect.hclust” function from the vegan package in R [45].

To evaluate the effect of *Marenzelleria* spp. biomass (Pol) on the composition differences between Communities 1 and 2, a Permutational Multivariate Analysis of Variance (PERMANOVA) was conducted using the “adonis2” function from the vegan package in R [45]. The Bray–Curtis dissimilarity matrix was used as the response variable with Pol as the explanatory variable. The analysis included 999 permutations to assess the significance of the *Marenzelleria* spp. biomass on the community structure.

Principal Component Analysis (PCA) was performed to explore relationships among environmental variables and zoobenthos biomass. The PCA was conducted using the “prcomp” function in R [44] with scaling and centering of the data. The first two principal components (PC 1 and PC 2) were selected for visualization, explaining 41.8% of the total variance. To visualize the results, a biplot was generated using the “fviz_pca_biplot” function from the factoextra package in R [46]. Sample points were color-coded according to their respective community types, and 95% confidence ellipses were added for each group. Variables were represented by vectors indicating their direction and contribution to the principal components. All visualizations were customized using the ggplot2 package in R [47] to enhance readability.

To assess the differences in environmental variables between communities, a one-way analysis of variance (ANOVA) was conducted for each variable. The response variables were normalized by centering and scaling the data before analysis. ANOVA was applied with community type as the grouping factor. For each variable, post hoc pairwise comparisons between communities were performed using Tukey’s Honestly Significant Difference (HSD) test to identify which specific groups differed significantly. The analysis was carried out in R using the “aov” function [44] for ANOVA and the “TukeyHSD” function [44] for post hoc comparisons. Significance was determined at a 95% confidence level, and *p*-values were adjusted to account for multiple comparisons.

Finally, to evaluate the impact of various environmental factors on the biomass of *Marenzelleria* spp., a one-way ANOVA was performed using the “aov” function in R [44]. The model included the following variables: Sal, Temp, pH, Eh, Turb, Depth, CHL, PP, MN, PP to MN ratio, SMe, and SMg. The total variance explained by the model was calculated with significant factors identified and their individual contributions to the total variance outlined.

All data and the full script of statistical analyses can be found in the Supplementary Materials.

## 3. Results

**Figure 2:**
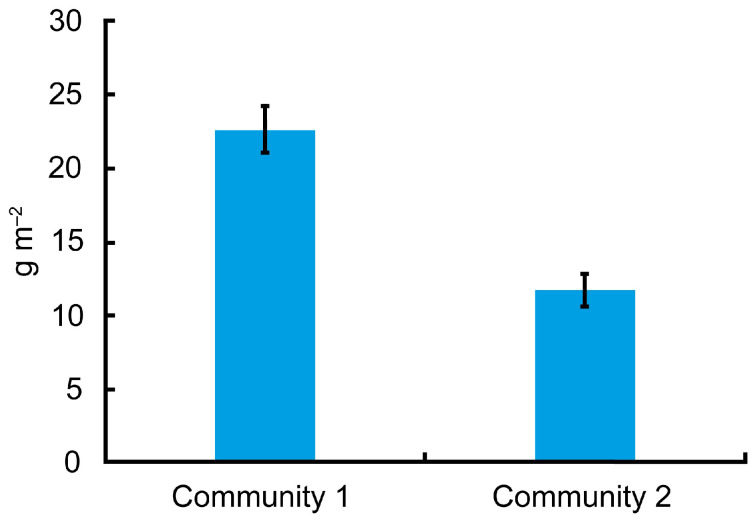
The total biomass of zoobenthos at high (>3.84 g/m^2^, Community 1) and low (<3.84 g/m^2^, Community 2) biomass of alien polychaetes *Marenzellaria* spp.

The average biomass of *Marenzelleria* spp. at the sampling stations throughout the study period was 3.84 ± 1.31 g m^−2^. A notably high biomass and abundance of *Marenzelleria* spp. was observed in the first years of the research at deep sampling stations (>20 m depth), while low biomass and abundance were recorded in 2021–2023 at both deep and shallow sampling stations (<20 m depth) (Table 1 and Table 2). Overall, their average biomass in the entire middle part of the estuary decreased by nearly two orders of magnitude during the study period. The maximum biomass and abundance of polychaetes were usually found at deep-water stations 4, 5 and 6, whereas the minimum biomass and abundance were more often observed at shallow-water stations 1, 2, 3 and 7. In contrast, the total biomass of zoobenthos exhibited relatively minor fluctuations during this period. The maximum average zoobenthos biomass across all sampling stations was recorded in 2017 at 21.40 ± 5.17 g m^−2^, while the minimum average biomass of 4.74 ± 1.26 g m^−2^ occurred in 2023. The maximum average abundance of zoobenthos across all sampling stations was found in 2016 and amounted to 17,909 ± 5183 ind. m^−2^, and the minimum average abundance, 4467 ± 607 ind. m^−2^, was recorded in 2021. At sampling stations where the biomass of polychaetes fell below than the average value of 3.84 g m^−2^, the total biomass of zoobenthos was lower compared to stations with polychaete biomass above the average (Figure 2).

**Figure 3:**
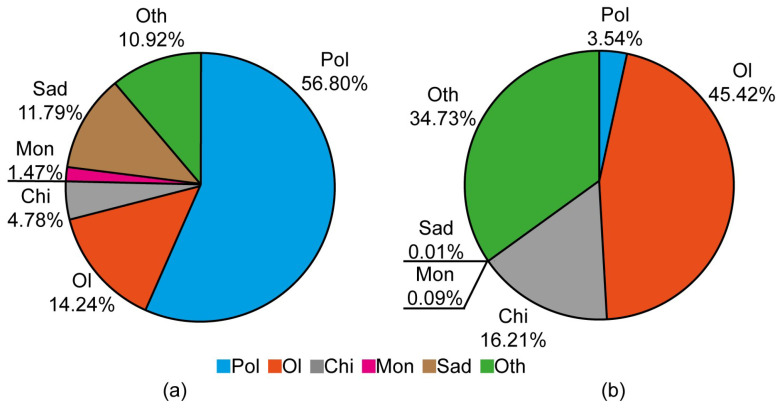
The portion of different groups in total biomass of zoobenthos at high (>3.84 g m^−2^, Community 1) (**a**) and low (<3.84 g m^−2^, Community 2) (**b**) biomass of alien polychaetes *Marenzellaria* spp. Pol—*Marenzellaria* spp., Ol—Oligochaeta, Chi—*Chironomus plumosus*, Sad—*Saduria entomon*, Mon—*Monoporeia affinis*, Oth—other species.

When the biomass of polychaetes at sampling stations exceeded the average value, they contributed significantly to the total biomass of zoobenthos (Figure 3a). The native species *Potamothrix hammoniensis* (Michaelsen), *Limnodrilus hoffmeisteri* Claparède (Oligochaeta), the predatory isopod *Saduria entomon* (Linnaeus), and the larvae of *Chironomus plumosus* (Linnaeus) (*Diptera, Chironomidae*) had much lower biomass values. The indigenous amphipod *Monoporeia affinis* (Lindstrom) constituted only about 1% of the total zoobenthos biomass. A different structure of the zoobenthos community was observed at a low biomass (˂3.84 g m^−2^) of alien polychaetes, where their contribution to total zoobenthos biomass averaged just 4%, and oligochaetes dominated (Figure 3b). The larvae of *Ch. plumosus* also played a prominent role in the biomass of benthic macroinvertebrates. At these stations, *S. entomon* was usually absent, and the biomass of *M. affinis* was very low. Occasionally, *Macoma balthica* (Linnaeus) was present in this community, significantly contributing to the zoobenthos biomass in those instances. Overall, the biological diversity of zoobenthos was lower when alien polychaetes were less abundant compared to periods of high abundance.

**Figure 4:**
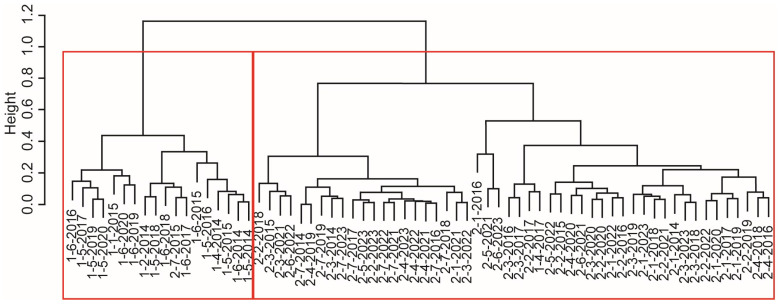
Dendrogram illustrating the clustering of samples based on Bray–Curtis dissimilarity using the Ward’s minimum variance method with squared Euclidean distance. The dendrogram represents hierarchical clustering with labeled by combinations of community–station–year.

The dendrogram resulting from Hierarchical Clustering Analysis based on the Bray–Curtis dissimilarity index (Figure 4) clearly illustrates the clustering of communities, sampling stations, and years. Two major clusters were identified, marked by red boxes, suggesting distinct groupings of communities. The close proximity of certain communities from different stations or years suggests ecological similarities, while the separation of others highlights significant differences in community composition. The first cluster corresponds to Community 1, primarily comprising deep-water stations in the western part of the estuary (stations 5 and 6). However, some shallow-water stations from the northern part were also included, for example station 1 in 2015 and station 2 in 2014, reflecting the early thriving populations of alien polychaetes. The second cluster aligns to Community 2, mainly comprising shallower stations, but also including some deep-water stations, like station 6 from 2022 and station 5 from 2023, during periods when the populations of alien polychaetes were declining (Figure 4).

The results of the PERMANOVA analysis demonstrated that the biomass of *Marenzelleria* spp. had a statistically significant effect on the Bray–Curtis dissimilarity between communities (F = 49.43, *p* = 0.001). The model accounted for 42.82% of the total variance in dissimilarity (R^2^ = 0.43), indicating that the biomass of polychaetes explains a substantial proportion of the variation in community structure. The remaining 57.18% of the variation was attributed to residuals. The low *p*-value (*p* = 0.001) confirms the robustness of this effect, suggesting that differences in biomass between communities significantly contribute to their ecological differentiation (Table 3).

**Figure 5:**
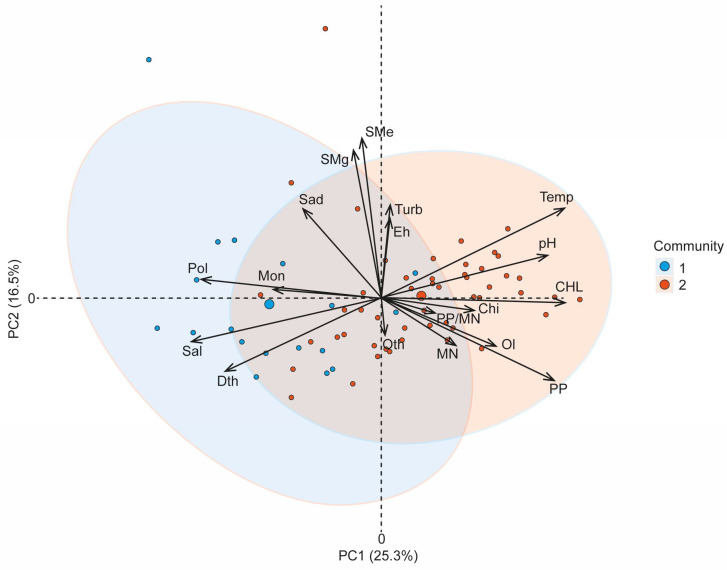
Principal Component Analysis (PCA) biplot showing the distribution of samples based on environmental variables. Points represent individual samples, colored according to the “Community” variable with high (>3.84 g m^−2^, 1) and low (<3.84 g m^−2^, 2) biomass of *Marenzelleria* spp., with ellipses indicating 95% confidence intervals for each community. The large blue and red dots are the centers of the ellipses for each data set. Arrows indicate the direction and strength of the contribution of variables to the principal components. PC1 and PC2 explain 25.3% and 16.5% of the total variance, respectively. Sal is water salinity (PSU); Temp—water temperature (°C); Dth—depth (m); Turb—water turbidity (NTU), pH—hydrogen potential; Eh—Oxidation/Reduction Potential (mV); SM—concentration of particulate suspended matter above (e) and below (g) thermocline; CHL—chlorophyll *a* concentration in water (mg m^–3^); PP—plankton primary production (gC m^−2^ d^–1^); MN—rate of mineralization of organic matter in water column (gC m^−2^ d^–1^); PP/MN—PP to MN ratio. The decoding of symbols corresponding to groups and species of zoobenthos is given in the caption to Figure 3.

The PCA biplot (Figure 5) displays the first two principal components (PC1 and PC2), which together explain 41.8% of the total variance (PC1: 25.3%, PC2: 16.5%). Each point represents a sample with colors distinguishing Community 1 (blue) from Community 2 (red). Environmental variables are represented as vectors. The PCA biplot shows that Community 1 and Community 2 are clearly separated, as indicated by the distinct ellipses, suggesting that these communities are influenced by different sets of environmental factors. The length and direction of the arrows represent the contribution and influence of each environmental variable. For example, variables such as Pol, Dth, and Sal are strongly associated with Community 1, while CHL, Temp, and pH are more closely related to Community 2.

PC 1 captures a gradient of variables related to salinity and zoobenthos biomass with Pol and Sal contributing significantly to this principal component. In contrast, PC 2 captures variability related to chlorophyll and pH levels that differentiate the two communities. These results highlight the significant role of environmental factors in shaping community structure with Pol, Sal, and Dth being key differentiators for Community 1, while CHL and pH are more influential in Community 2. This is consistent with findings from PERMANOVA analysis, which demonstrated significant effects of Pol on community dissimilarity (Table 3).

The biomass of *Marenzellaria* spp. is strongly correlated with PC 1, as indicated by the long vector pointing to the left. This suggests that Community 1 (blue) is characterized by higher polychaete biomass, which is also associated with environmental variables such as Sal and Dth. During the study years, the salinity of near-surface waters ranged from 1 to 3 PSU, while that of bottom waters ranged from 1 to 5 PSU. The clustering of Community 1 samples along PC 1 indicates that in the Neva estuary, *Marenzelleria* spp. and *M. affinis* (Mon) thrived in biotopes with high salinity and water depth.

In contrast, the biomass of chironomid larvae (Chi) and oligochaetes (Ol) shows a stronger association with PC 2, which was positively influenced by factors such as chlorophyll (CHL), pH, and temperature (Temp). These zoobenthic groups are more prevalent in Community 2 (red), suggesting that this community is better adapted to environments with lower salinity and higher productivity, as indicated by CHL, PP and pH. The biomass of *S. entomon* (Sad) is positioned centrally between PC 1 and PC 2, implying that this species is less strongly influenced by the environmental gradients captured by these components. The vector for other benthic species (Oth) is relatively short, indicating a lower contribution of these species to the overall variability explained by the PCA.

This distribution of benthic biomass vectors suggests that Community 1 is primarily structured by environmental factors such as salinity and depth, favoring *Marenzellaria* spp. and *M. affinis*. Conversely, Community 2 appears to be structured by productivity and water quality parameters (e.g., chlorophyll and pH), supporting chironomids and oligochaetes (Figure 5). These findings align with the previously observed clustering patterns (Figure 4) and provide further evidence of the ecological drivers influencing community composition.

The results of Tukey’s HSD test (Table 4) revealed significant differences in the mean biomass of *Marenzellaria* spp. between communities, with a mean difference of −0.746 (95% CI: −0.374 to −0.627, *p* < 0.0001), indicating that polychaete biomass is significantly higher in Community 1 than in Community 2. A smaller yet still significant difference was observed for *M. affinis* (mean difference = −0.066, *p* < 0.0001) and *S. entomon* (mean difference = −0.099, *p* = 0.0376).

The environmental variables salinity and depth showed the strongest significant positive differences with salinity having a difference of −0.099 (*p* = 0.0129) and depth a difference of −0.193 (*p* < 0.0001). These variables appear to be strongly associated with changes in community structure. The temperature was significantly lower in habitats occupied by Community 1 compared to habitats occupied by Community 2 (difference = 0.270, *p* < 0.0001), suggesting that the composition of Community 1 might be linked to cooler conditions in the water area or period of investigation. pH levels were slightly but statistically significantly lower in habitats with Community 1 than in habitats with Community 2 (difference = 0.009, *p* = 0.0011), indicating minor differences in acidity between the habitats occupied by these two communities.

Chlorophyll levels were also lower in habitats with Community 1 than in those with Community 2 (difference = 0.149, *p* = 0.0008), suggesting that the higher polychaete abundance was associated with lower chlorophyll concentrations at the stations. Plankton primary production (PP) exhibited significant but smaller differences between the habitats occupied by the two communities with PP showing a difference of 0.066 (*p* = 0.0105).

Several other variables, including the biomasses of oligochaetes and chironomids, did not show significant differences between communities with *p*-values exceeding 0.05. Environmental variables such as oxidation-reduction potential, turbidity, and suspended matter concentration did not differ significantly between biotopes inhabited by communities with high and low biomass of alien polychaetes (*p* > 0.4).

Results of the Analysis of Variance (ANOVA) (Table 5) indicate that several environmental variables significantly contributed to explaining the variation in *Marenzellaria* spp. biomass across the studied communities. Salinity explained 11.53% of the total variance in polychaete biomass and had a significant effect (F = 16.93, *p* = 0.00013), suggesting that salinity was one of the major drivers of polychaete biomass distribution in the studied communities. Temperature was another important factor, explaining 13.13% of the variance (F = 19.28, *p* = 0.00005). This indicates that polychaete biomass tended to vary significantly with temperature fluctuations across different habitats. Turbidity had the largest individual effect, accounting for 14.20% of the variance in polychaete biomass (F = 20.84, *p* = 0.00003). The strong influence of turbidity suggests that water transparency or particulate matter could be influencing polychaete abundance either through habitat modification or resource availability. The concentration of suspended matter above the thermocline (F = 8.55, *p* = 0.00501) was also a significant variable with turbidity and suspended matter together explaining 20.02% of variance. Water depth explained 6.80% of the variance (F = 9.97, *p* = 0.00258), emphasizing its role in the formation of polychaete populations; these animals in the Neva estuary usually predominated in deep-water habitats. Redox Potential (Eh) explained 4.33% of the variance (F = 6.36, *p* = 0.01458), suggesting that sediment oxygenation conditions could also influence polychaete populations.

Several other factors, such as the rate of mineralization of organic matter (MN, 2.73% variance explained, *p* = 0.05030), chlorophyll *a* concertation (CHL, 1.77% variance explained, *p* = 0.12263) and plankton primary production (PP, 1.36% variance explained, *p* = 0. 16325) showed weaker effects with marginal significance. Factors like pH and PP/MN had no significant effects on polychaete biomass (*p* > 0.3), with pH and PP/MN explaining only 0.70% and 0.14% of the variance, respectively. Finally, residual variance accounted for 37.47% of the total variance, suggesting that other unmeasured variables or random variation also contributed to the observed variation in polychaete biomass.

## 4. Discussion

Although it is generally accepted that invasive species usually have negative impacts on recipient ecosystems, there are a few documented cases of positive effects from their invasions (e.g., [5,48,49]). Therefore, the consequences of an invasion should always be carefully studied, taking into account the traits of both invaders and resident species as well as their environmental context [13]. This is also important for determining the threshold values for the risk of introduction of certain taxonomic groups into water bodies in certain climate zones. This ecological knowledge, combined with the invasion risk rating developed from the recent global screening of potential invaders (over 800 species of aquatic organisms) [50], will help decision-makers decide which species should be restricted or banned for import or sale for economic purposes and which should be included in national invasive species monitoring programs.

There are numerous well-documented examples of extinctions of native species following the introduction of predatory NISs, but cases in which the extinction of native species can be explained by competition from NISs are quite rare [15,51]. It follows that the introduction of an alien species does not necessarily lead to a decline in biodiversity. If sufficient resources are available, an invasive species may well coexist with native species, potentially increasing the species richness of the community [51]. In addition, the temporary depletion of local communities can free up additional resources for NISs, facilitating their successful invasions, as observed in fish communities [16].

In the case of the Neva estuary, zoobenthos communities with low biomass of the alien polychaetes *Marenzelleria* spp. differed with high confidence from zoobenthos communities with a high biomass of these alien species (Figure 2, Figure 3 and Figure 4 and Table 3). It is noteworthy that in Community 1, the indigenous crustaceans *S. entomon* and *M. affinis* played a perceptible role in the biomass of zoobenthos (Figure 3). These species, which are widespread in the Baltic Sea, dominated the Neva estuary’s zoobenthos until the mid-1990s [35,52]. However, their biomass declined significantly following a series of bottom hypoxia events in the mid-2000s. When oxygen conditions at the bottom improved in the late 2000s, zoobenthos biomass was recovered due to an explosive growth in populations of alien *Marenzelleria* spp., which subsequently came to dominate the estuary’s zoobenthos, especially at deep-water stations. Oligochaetes became the subdominant group of bottom macroinvertebrates. This structure of zoobenthos in the Neva estuary remained until the mid-2010s [14] after which a rapid decline in the biomass of alien *Marenzelleria* spp. began (Table 1). Simultaneously with the decline of polychaetes, the biomass of indigenous crustaceans, *S. entomon* and *M. affinis*, decreased even more. Their biomass in zoobenthos communities with a low biomass of alien polychaetes was significantly lower than in those with a high biomass of these NISs (Table 4, Figure 5).

Principal Component Analysis showed that the requirements for environmental factors in alien polychaetes and local benthic crustaceans largely coincide. Both groups showed increased biomass in deeper, brackish water biotopes characterized by low temperatures and primary productivity. The vectors representing changes in the biomass of *M. affinis* and *Marenzelleria* spp. closely aligned within the space of the studied environmental variables. Specifically, depth, temperature, and salinity positively influenced both species, while chlorophyll concentration exhibited a negative effect. Notably, primary production and chlorophyll levels in the water were the main environmental variables negatively affecting the biomass of *S. entomon* (Figure 5). This finding is consistent with the native habitat of *Marenzelleria arctia*, which thrives in clear, cold-water biotopes of northern estuaries alongside crustaceans such as *M. affinis* and *S. entomon* [53]. Previous studies have also demonstrated that depth and low temperatures positively influence the abundance of *Marenzelleria* spp. across various regions of the Baltic Sea [10,23]. *M. affinis*, a glacial relic, also prefers low temperatures, as its gonads and embryos develop more slowly when water temperatures exceed 10 °C [54]. In contrast to polychaetes and benthic crustaceans, the biomass of oligochaetes and chironomids increased in shallow, warmer water of the Neva estuary (Figure 5). However, these groups did not show significant differences in zoobenthos communities with either low or high biomass of *Marenzelleria* spp. (Table 4).

Invasive species pass through both biotic and abiotic filters during invasion [15]. Biotic filters include food availability, spatial resources competition with indigenous species, and predation pressure. As previously noted, the explosion in *Marenzelleria* abundance coincided with the decline of native species due to a series of hypoxic events in the Neva estuary, which likely freed up essential resources for polychaete population development. Their planktonic larvae and the bottom countercurrent directed from the sea to the middle part of the estuary appear to have facilitated rapid settlement in biotopes previously occupied by local species.

Both amphipods and alien polychaetes are capable of occupying similar habitats in the Baltic Sea, and the fact that both *Monoporea* and *Marenzelleria* are deposit feeders may lead to competition for food [55]. However, in the context of the Neva estuary, their feeding modes do not completely overlap. Stable isotope analysis (SIA) has shown that *Marenzellaria* primarily utilize allochthonous carbon from the catchment area, whereas autochthonous carbon produced by phytoplankton plays a significant role in the diet of *M. affinis* [38]. Oligochaetes also mainly use allochthonous carbon, but not from the surface of bottom sediments like *Marenzellaria* spp., but rather from deep layers. This likely reduces competition between these groups of worms for food. Additionally, SIA indicates that invertebrate predators and benthic fish practically do not consume these alien polychaetes, which hide from predators in deep burrows [14].

The results of the one-way analysis of variance showed that six environmental factors can be considered as components of the abiotic filter for the invasion of *Marenzellaria* spp. into the Neva estuary with a high degree of confidence (Table 5). This generally confirms the results of Principal Component Analysis (Figure 5). Among these six factors, temperature, salinity, and water turbidity had the strongest and most reliable influence on the biomass of alien polychaetes. However, the last of these factors rather reflects their preference of deep-water biotopes with low turbidity (Figure 5). This is also supported by a significant negative relationship between *Marenzellaria* biomass and the concentration of suspended matter in the layer above thermocline (Table 5). Biotope depth was also a significant factor influencing the biomass of alien polychaetes in the Neva estuary (Table 5) as well as in other parts of the Gulf of Finland [10]. Although we did not observe events of benthic hypoxia during our studies, a significant relationship between the value of Eh and the biomass of *Marenzellaria* spp. indicated the influence of oxygen conditions on these polychaetes. Previous studies have shown that *Marenzelleria* species can tolerate low oxygen levels in the water [56,57], which likely provides them with a competitive advantage over some indigenous species in the benthic communities of the Baltic Sea. However, hypoxic events have been known to reduce the abundance of these worms in deeper areas, and their density has shown a significant positive correlation with dissolved oxygen levels [10,31].

Residual variance accounted for 37.47% of the total variance, suggesting that unmeasured variables or random variation may also contribute to the observed variation in polychaete biomass. Vivó-Pons et al. [23] highlight various direct and indirect links between abiotic and biotic variables that influence the relationship between *Marenzelleria* and native species communities. Additionally, the dynamics of environmental conditions may play a critical role in the survival of their planktonic larvae, which were not considered in this study. For example, the biomass of marine and brackish mesozooplankton in the Baltic Sea, including *Marenzellaria* larvae, is significantly affected by changes in the North Atlantic Oscillation [58]. In recent years, shifts in this oscillation have led to substantial losses in biomass and functional biodiversity among some mesozooplankton groups.

Another crucial environmental factor affecting biomass in the Neva estuary is the salinity at the bottom (Figure 5, Table 5). Previous research has indicated that *M. viridis* larvae are well adapted to brackish water environments; however, the oligohaline region may represent the boundary for the generative distribution of *Marenzellaria* [25]. Laboratory experiments have shown that *Marenzellaria viridis* (Verrill) larvae can survive brief salinity drops to 1‰ and, after reaching or passed the four-setiger stage, can even develop into benthic juveniles at a salinity of 3.5‰ [59]. This adaptability allows them to colonize oligohaline regions during their early life stages. However, prolonged decreases in water salinity can inhibit metamorphosis and result in larval mortality [59]. Fluctuations in weather conditions and changes in the phases of teleconnection patterns have a significant impact on the state of the environment in the Neva estuary [32,33]. The increased frequency of rainy weather in the late 2010s may have caused periodic decreases in bottom water salinity, potentially slowing the replenishment rate of *Marenzelleria* populations with young individuals and adversely affecting their biomass in the estuary.

Invasive biology primarily focuses on the problems of the introduction of NISs into new habitats and their effects on native communities (e.g., [3,7,15,60]). However, it should be taken into account that both negative and positive impacts on ecological systems are most pronounced during periods of peak NIS abundance. Understanding the environmental conditions that facilitate the maximum abundance of an alien species is therefore essential. It is generally accepted that the dynamics of NIS abundance typically follow a “boom and bust” pattern characterized by an initial sharp increase in numbers followed by a subsequent decline. However, this model has numerous exceptions. Often, the introduction of an alien species into a new habitat and its subsequent boom phase can be separated by a significant time lag. For example, *Marenzelleria* spp. were first discovered in the Baltic Sea in 1985, yet a marked increase in their abundance across most areas of the sea occurred much later in the mid-2000s [10]. Additionally, the boom phase can persist for extended periods; high biomasses of the alien crustacean *Cercopagis pengoi* in the eastern Gulf of Finland have been recorded from the late 1990s to the present day [19]. These observations suggest that the introduction of NISs does not necessarily occur under the most optimal conditions for it. However, if such conditions subsequently arise, a boom phase is observed in population dynamics, continuing until environmental conditions deteriorate and a bust phase ensues.

The decline in *Marenzelleria* biomass was likely driven by a deterioration in living conditions for zoobenthos in the Neva estuary, beginning in the latter half of the 2010s. This is confirmed by the simultaneous decrease in the biomass of bottom crustaceans. In a zoobenthos community with low biomass of polychaetes, opportunistic freshwater oligochaete species and chironomid larvae, common in polluted waters, began to dominate sharply (Figure 3b). This community exists with high significance at higher temperatures, low salinity and more eutrophic waters (Table 4). The changes in environmental conditions for zoobenthos are probably associated with both climate change and increased anthropogenic impacts on the estuarine ecosystem during this period [32,33,61]. Given that climate models predict future increases in precipitation and temperature in the northern hemisphere, declines in populations of marine species, including NISs, in boreal and possibly Arctic estuaries are highly probable. More detailed studies are needed to elucidate the responses of alien species to environmental variables to better understand their dynamics in different northern hemisphere estuarine waters. This is particularly important in the case of *Marenzelleria* spp., which have been shown to inhibit the eutrophication process in the coastal waters of the Baltic Sea [11,12]. An important aspect of the problem is that existing monitoring systems for NISs do not have sufficient data on their impacts in different sub-basins and on local species communities [62].

## 5. Conclusions

Identifying key factors associated with invasion hotspots allows the comparison and prioritization of NISs according to their potential invasiveness and the potential harm they may cause to recipient communities under current and future climate conditions. The study of the dynamics of biomass of alien polychaetes and the factors influencing their abundance in the Neva estuary demonstrates that the invasiveness and harmfulness of NISs can vary significantly. The success of *Marenzelleria* populations in the estuary appears to be more closely related to their ability to quickly colonize biotopes freed from native zoobenthos rather than to direct competition with local species. Notably, the main factors influencing the abundance of alien polychaetes and native crustaceans are largely similar. Both groups show a negative relationship with increasing temperature, primary production, and algae biomass. If we assume that further climate warming may result in elevated temperatures and increased eutrophication in the coastal zone of the Baltic Sea, then the future forecast for an increase in the biomass of these groups of macroinvertebrates in the estuaries is highly unlikely. Conversely, more frequent occurrences of bottom hypoxia, driven by either climate change or intensified anthropogenic impacts, are likely to favor the expansion of areas occupied by *Marenzelleria* spp., owing to their rapid colonization of disturbed biotopes. These factors must be considered when assessing the risk of further expansion of these alien polychaetes. Therefore, accumulating information on the dynamics of specific alien and native species in particular habitats, as well as the key ecological factors influencing their dynamics, could be critical for enhancing NISs monitoring systems.

## Figures and Tables

**Table 1:** Changes in the average, maximum and minimum biomass of alien polychaetes *Marenzelleria* spp. at shallow (<20 m depth) and deep (>20 m depth) sampling stations in the middle part of the Neva estuary in 2014–2023. AB—average biomass; MaxB—maximum biomass; MinB—minimum biomass.

Years	Shallow Stations			Deep Stations		
	AB, (g m^−2^)	MaxB, (g m^−2^)	MinB, (g m^−2^)	AB, (g m^−2^)	MaxB, (g m^−2^)	MinB, (g m^−2^)
2014	1.61	4.08	0.20	16.67	20.06	12.72
2015	4.83	12.28	1.19	28.83	34.02	23.66
2016	0.25	0.50	0.04	12.82	26.24	2.06
2017	0.25	0.55	0.00	10.80	21.16	5.12
2018	0.11	0.36	0.02	5.68	7.98	1.94
2019	0.90	1.94	0.36	7.84	9.42	6.26
2020	0.46	1.09	0.06	5.51	11.40	0.06
2021	0.08	0.24	0.00	0.36	0.62	0.03
2022	0.16	0.41	0.02	0.14	0.41	0.02
2023	0.20	0.40	0.00	0.33	0.75	0.00

**Table 2:** Changes in the average, maximum and minimum abundance of alien polychaetes *Marenzelleria* spp. at shallow (<20 m depth) and deep (>20 m depth) sampling stations in the middle part of the Neva estuary in 2014–2023. AN—average abundance; MaxN—maximum abundance; MinN—minimum abundance.

Years	Shallow Stations			Deep Stations		
	AN, (ind. m^−2^)	MaxN, (ind. m^−2^)	MinN, (ind. m^−2^)	AN, (ind. m^−2^)	MaxN, (ind. m^−2^)	MinN, (ind. m^−2^)
2014	148	400	20	2527	2880	2300
2015	360	720	120	8670	9080	8260
2016	75	140	20	2473	4520	400
2017	165	440	0	5987	8180	2740
2018	180	520	20	1720	2440	420
2019	780	2080	140	4640	5560	3720
2020	145	300	60	1087	1920	100
2021	20	40	0	273	420	60
2022	102	180	13	807	1820	80
2023	82	117	26	230	351	0

**Table 3:** Results of PERMANOVA analysis evaluating the effect of *Marenzelleria* spp. biomass on Bray–Curtis dissimilarity between communities.

Source of Variation	Degrees of Freedom	Sum of Squares	R^2^	F-Value	*p*-Value
Model	1	0.750	0.43	49.43	0.001
Residuals	66	1.002	0.57		
Total	67	1.752	1		

**Table 4:** Results of Tukey’s Honestly Significant Difference (HSD) test comparing environmental variables across different community types with low (Community 1, biomass < 3.84 g m^−2^) and high (Community 2, biomass > 3.84 g m^−2^) biomass of *Marenzellaria* spp. Each variable was analyzed using a one-way ANOVA, which was followed by post hoc Tukey HSD tests to identify pairwise differences between communities. The table presents the mean differences between community pairs (diff), along with the lower (lwr) and upper (upr) bounds of the 95% confidence intervals, and the adjusted *p*-values (*p*-value). Significant differences (*p* < 0.05) are indicated in bold, highlighting where community types differ for each environmental variable. The decoding of symbols corresponding to environmental variables is given in the caption to Figure 5.

Variable	Diff	lwr	upr	*p*-Value
The mean value of the variable in Community 2 is statistically significantly lower than in Community 1				
**Pol**	−0.746	−0.374	−0.627	**<0.0001**
**Mon**	−0.060	−0.082	−0.038	**<0.0001**
**Sad**	−0.099	−0.192	−0.006	**0.0376**
**Dth**	−0.193	−0.262	−0.125	**<0.0001**
**Sal**	−0.089	−0.158	−0.019	**0.0129**
The mean value of the variable in Community 2 is statistically significantly higher than in Community 1				
**Temp**	0.270	0.165	0.375	**<0.0001**
**pH**	0.009	0.003	0.014	**0.0011**
**CHL**	0.149	0.064	0.234	**0.0008**
**PP**	0.061	0.015	0.107	**0.0105**
Comparison of mean values between Community 1 and Community 2, showing no statistically significant differences for the listed variables				
Chi	0.119	−0.008	0.247	0.0661
Ol	0.136	−0.023	0.295	0.0899
Oth	−0.106	−0.277	0.064	0.2176
Eh	−0.008	−0.077	0.060	0.8060
Turb	0.009	−0.122	0.141	0.8882
SMe	0.017	−0.078	0.112	0.7230
SMg	−0.009	−0.096	0.078	0.8367
MN	0.053	−0.025	0.130	0.1787
PP/MN	0.019	−0.023	0.062	0.3743

**Table 5:** Results of the one-way analysis of variance (ANOVA) for the effect of environmental factors on the response variable *Marenzelleria* spp. biomass. Factors with *p*-values less than 0.05 were considered statistically significant and were highlighted in bold. The total variance explained by the model and the residual variance are also included. The decoding of symbols corresponding to environmental variables is given in the caption to Figure 5.

Variable	Degrees of Freedom	Sum of Squares	Mean of Squares	F-Value	*p*-Value	Fraction of Variance
**Sal**	**1**	**1.013**	**1.013**	**16.93**	**0.00013**	**11.53**
**Temp**	**1**	**1.154**	**1.154**	**19.28**	**0.00005**	**13.13**
**Turb**	**1**	**1.248**	**1.248**	**20.84**	**0.00003**	**14.20**
**Dth**	**1**	**0.597**	**0.597**	**9.97**	**0.00258**	**6.80**
**SMe**	**1**	**0.512**	**0.512**	**8.55**	**0.00501**	**5.82**
**Eh**	**1**	**0.381**	**0.381**	**6.36**	**0.01458**	**4.33**
MN	1	0.240	0.240	4.00	0.05030	2.73
CHL	1	0.156	0.156	2.60	0.11263	1.77
PP	1	0.120	0.120	2.00	0.16325	1.36
pH	1	0.061	0.061	1.02	0.31648	0.70
PP/MN	1	0.013	0.013	0.21	0.64764	0.14
SMg	1	0.000	0.000	0.000	0.98634	0.20
**Residuals**	55	3.293	0.0599			**37.47**

## Data Availability

The original contributions presented in the study are included in the article/Supplementary Materials. Further inquiries can be directed to the corresponding author.

## Supplementary Materials

The following supporting information can be downloaded at https://www.mdpi.com/article/10.3390/biology13120974/s1, Supplementary Materials 1: Table S1—Biomass of zoobenthos in the Neva estuary. Each number is the average of three replicates. Table S2—Environmental variables at benthic sampling stations in the Neva River estuary. Supplementary Materials 2: Script in the R programming language for data analysis.
