# Supplementary material for: Dynamics of Marenzelleria spp. Biomass and Environmental Variability: A Case Study in the Neva Estuary (The Easternmost Baltic Sea)
Source: Biology (Basel). 2024 Nov 26;13(12):974. doi: 10.3390/biology13120974 (PMC11673274; doi:10.3390/biology13120974)
Supplement: Supplementary file 1 [file biology-13-00974-s001.zip › Supl2.pdf]

Article: Dynamics of *Marenzelleria* spp. biomass and environmental variability. A case study in the Neva estuary (the easternmost Baltic Sea)

The authors: Sergey M. Golubkov[1*], Mikhail S. Golubkov[1].

1 – Zoological Institute of Russian Academy of Sciences, St.-Petersburg, Russian Federation.

*Corresponding author e-mail: golubkov@zin.ru

Mikhail S. Golubkov e-mail: golubkov_ms@mail.ru

Script in the R programming language for data analysis:

```
> data <- Benthos

>library(vegan)

> data_no_community_year_station <- data[, !(names(data) %in% c("Community", "Year", "Station"))]

> bray_curtis_index <- vegdist(data_no_community_year_station, method = "bray")

> bray_curtis_matrix <- as.matrix(bray_curtis_index)

> community_station_year_labels <- paste(data$ Community, data$Station, data$Year, sep = " - ")

>  dend <- hclust(bray_curtis_index, method = "ward.D2")

> plot(dend,

      main = "Cluster Dendrogram of Bray-Curtis Distance",

      labels = community_station_year_labels,

      cex = 1.5,

      cex.axis = 1.5,

      cex.lab = 1.5)

> rect.hclust(dend, 2)

> permanova_result <- adonis2(bray_curtis_index ~ Pol, data = data)

> print(permanova_result)

> library(factoextra)

> library(ggplot2)

>data2 <- data.frame(

   Community = data$ Community,

   Pol = data$Pol,

    Ol = data$Ol,

   Chi = data$Chi,

   Mon = data$Mon,
```

```
 Sad = data$Sad,
 Oth = data$Oth,
Sal = Benthos_env$Sal,
Temp = Benthos_env$Temp,
pH = Benthos_env$pH,
Eh = Benthos_env$Eh,
Turb = Benthos_env$Turb,
 Dth = Benthos_env$Dth,
 SMe = Benthos_env$SMe,
 SMg = Benthos_env$SMg,
 CHL = Benthos_env$CHL,
 PP = Benthos_env$PP,
 MN = Benthos_env$MN,
PPMN = Benthos_env$PPMN)
> data2.pr <- prcomp(data2 [c(2:19)], center = TRUE, scale = TRUE)
> data2$Community <- factor(data2$Community)
> fviz_pca_biplot(data2.pr, geom.ind = "point", pointshape = 21, pointsize = 2,
            fill.ind = data2$Community,
            col.ind = "black",
            palette = "jco",
            addEllipses = TRUE,
            label = "var",
            col.var = "black",
            legend.title = "Community",
            labelsize = 5) +
   theme(
     axis.title = element_text(size = 14),
```

```
        axis.text = element_text(size = 12),
        legend.title = element_text(size = 12),
        legend.text = element_text(size = 10)
    )
> variables <- setdiff(names(Benthos_env), "Community")
> tukey_results <- lapply(variables, function(variable) {
    Benthos_env[[variable]] <- as.numeric(as.character(Benthos_env[[variable]]))
        aov_result <- aov(as.formula(paste0(variable, " ~ Community")), data = Benthos_env)
        tukey_result <- TukeyHSD(aov_result)
        return(tukey_result)
    })
> for (i in seq_along(variables)) {
        cat("Variable:", variables[i], "\n")
        print(summary(tukey_results[[i]]))
        cat("\n")
    }
> tukey_results
>data3 <- data.frame(
    Pol = data$Pol,
    Sal = Sal$ Benthos_env,
    Temp = Temp $ Benthos_env,
    pH = pH$Benthos_env,
    Eh = Eh$ Benthos_env,
    Turb = Turb$Benthos_env,
     Dth = Dth$Benthos_env,
     SMe = SMe$Benthos_env,
     SMg = SMg$Benthos_env,
```

```
    CHL = CHL$Benthos_env,

    PP = PP$Benthos_env,

    MN = MN$Benthos_env,

   PPMN = PPMN$Benthos_env,)

> aov_result <- aov(Pol ~ ., data = data3)

> summary(aov_result)

> summary_aov <- summary(aov_result)

> significant_factors <- summary_aov$coefficients[rownames(summary_aov) %in% c("Sal", "Temp", "pH",
"Eh", "Turb", "Dth", "CHL", "PP", "MN", "PPMN","SMe","SMg", "Residuals"),]

> total_variance <- sum(summary_aov[[1]]$'Sum Sq')

> results_table <- data.frame(Factor = character(), Variance_Explained = numeric(), stringsAsFactors =
FALSE)

> factor_names <- unique(rownames(summary_aov[[1]]))

> sum_sq <- summary_aov[[1]]$'Sum Sq'

> for (i in seq_along(factor_names)) {

   variance_explained <- (sum_sq[i] / total_variance) * 100

   results_table <- rbind(results_table, data.frame(Factor = factor_names[i], Variance_Explained =
variance_explained))

 }

> print(results_table)
```
