# Supplementary material for: Dynamics of Marenzelleria spp. Biomass and Environmental Variability: A Case Study in the Neva Estuary (The Easternmost Baltic Sea)
Source: Biology (Basel). 2024 Nov 26;13(12):974. doi: 10.3390/biology13120974 (PMC11673274; doi:10.3390/biology13120974)
Supplement: Supplementary file 1 [file biology-13-00974-s001.zip › Supl1.pdf]

Article: Dynamics of *Marenzelleria* spp. biomass and environmental variability. A case study in the Neva estuary (the easternmost Baltic Sea)

The authors: Sergey M. Golubkov[1*], Mikhail S. Golubkov[1].

1 – Zoological Institute of Russian Academy of Sciences, St.-Petersburg, Russian Federation.

*Corresponding author e-mail: golubkov@zin.ru

Mikhail S. Golubkov e-mail: golubkov_ms@mail.ru

Supplementary Table S1. Biomass of zoobenthos in the Neva estuary. Each number is the average of three replicates. The raw data were log-transformed (log_{10}) with an added constant of 2. Pol – biomass of *Marenzelleria* spp.; Ol – biomass of Oligochaeta; Chi – biomass of *Chironomus plumosus*; Mon – biomass of *Monoporeia affinis*; Sad – biomass of *Saduria entomon*; Oth – biomass of other species. Raw benthic biomass data were expressed as grams wet weight per square meter. Community: 1 – biomass of alien polychaetes *Marenzellaria* spp. > 3.84 g m^{-2}; 2 – biomass of alien polychaetes *Marenzellaria* spp. < 3.84 g m^{-2}.

Community	Year	Station	Pol	Ol	Chi	Mon	Sad	Oth
1	2014	2	0.78	0.74	0.31	0.30	0.30	0.33
1	2020	6	0.85	0.88	0.38	0.32	0.30	1.36
1	2017	4	0.85	1.21	1.28	0.30	0.30	0.41
1	2017	6	0.91	0.42	0.37	0.31	0.33	0.32
1	2019	6	0.92	0.82	0.47	0.35	0.30	0.65
1	2018	5	0.96	0.71	0.30	0.33	0.30	0.30
1	2018	6	1.00	0.40	0.31	0.54	0.30	0.35
1	2019	5	1.06	0.61	0.30	0.34	0.30	0.59
1	2016	6	1.08	0.42	0.41	0.52	0.30	1.11
1	2020	5	1.13	0.54	0.31	0.34	0.30	0.67
1	2015	1	1.15	1.27	0.39	0.30	0.30	0.47
1	2014	4	1.17	0.36	0.30	0.30	0.32	0.37
1	2014	6	1.28	0.45	0.31	0.30	0.30	0.30
1	2014	5	1.34	0.44	0.30	0.34	0.30	0.30
1	2017	5	1.36	0.64	0.30	0.41	0.30	0.89
1	2015	6	1.41	0.40	0.31	0.36	1.70	0.30
1	2016	5	1.45	0.59	0.30	0.50	0.64	0.30
1	2015	5	1.56	0.41	0.30	0.37	0.30	0.30
2	2014	7	0.30	0.30	0.30	0.30	0.30	0.30
2	2017	7	0.30	0.69	0.34	0.30	0.30	0.32
2	2019	4	0.30	0.30	0.30	0.30	0.30	0.30
2	2021	1	0.30	0.61	0.47	0.30	0.30	0.32
2	2023	4	0.30	0.49	0.34	0.30	0.30	0.32
2	2022	3	0.30	0.58	0.44	0.30	0.30	0.32
2	2018	7	0.31	0.39	0.43	0.36	0.30	0.31
2	2021	3	0.31	0.36	0.69	0.30	0.30	0.31
2	2018	1	0.31	1.01	0.58	0.30	0.30	0.38
2	2021	4	0.31	0.53	0.31	0.30	0.30	0.33
2	2022	6	0.31	0.48	0.59	0.30	0.30	0.30
2	2022	4	0.31	0.56	0.31	0.30	0.30	0.30
2	2016	3	0.31	1.17	0.92	0.30	0.30	0.40
2	2023	3	0.31	0.78	0.68	0.30	0.30	0.32
2	2022	2	0.31	1.11	0.56	0.30	0.30	0.35
2	2023	1	0.31	0.85	0.63	0.30	0.30	0.45
2	2018	3	0.31	0.72	0.68	0.30	0.30	0.33
2	2016	7	0.31	0.51	0.31	0.30	0.30	0.30

Supplementary Table S1. Biomass of zoobenthos in the Neva estuary. Each number is the average of three measurements (continued).

Community	Year	Station	Pol	Ol	Chi	Mon	Sad	Oth
2	2020	4	0.31	0.80	0.42	0.30	0.30	0.37
2	2021	2	0.31	0.91	0.54	0.30	0.30	0.33
2	2020	3	0.33	1.04	0.31	0.30	0.30	0.36
2	2022	7	0.33	0.60	0.31	0.30	0.30	0.32
2	2020	1	0.34	1.21	0.58	0.30	0.30	0.35
2	2014	3	0.34	0.46	0.34	0.30	0.30	0.35
2	2017	1	0.35	1.32	0.41	0.30	0.30	0.32
2	2017	3	0.35	1.12	1.05	0.30	0.30	0.33
2	2023	5	0.35	0.67	0.31	0.30	0.30	0.31
2	2021	7	0.35	0.54	0.31	0.30	0.30	0.32
2	2023	2	0.36	0.64	0.34	0.30	0.30	0.30
2	2018	2	0.37	0.52	1.02	0.30	0.30	0.39
2	2019	1	0.37	1.24	0.49	0.30	0.30	0.37
2	2016	1	0.38	1.31	0.83	0.32	0.30	2.23
2	2023	7	0.38	0.43	0.38	0.30	0.30	0.30
2	2022	1	0.38	0.89	0.48	0.30	0.30	0.40
2	2019	3	0.38	0.97	0.72	0.30	0.30	0.38
2	2021	5	0.39	0.56	0.30	0.31	0.30	1.36
2	2020	2	0.39	0.96	0.30	0.30	0.30	0.33
2	2016	2	0.40	1.07	0.46	0.30	0.30	0.35
2	2014	1	0.41	0.61	0.65	0.30	0.30	0.34
2	2017	2	0.41	1.07	1.36	0.30	0.30	0.42
2	2021	6	0.42	0.80	0.32	0.32	0.30	0.45
2	2023	6	0.44	0.65	0.31	0.30	0.30	0.90
2	2019	7	0.46	0.41	0.30	0.31	0.30	0.35
2	2015	3	0.50	0.42	0.68	0.30	0.30	0.37
2	2022	5	0.53	1.15	0.30	0.31	0.30	0.30
2	2018	4	0.60	0.94	0.75	0.30	0.31	0.32
2	2019	2	0.60	1.21	0.60	0.30	0.30	0.47
2	2016	4	0.60	1.03	0.61	0.31	0.30	0.34
2	2015	2	0.61	0.85	0.31	0.30	0.30	0.30
2	2015	7	0.76	0.45	0.36	0.30	0.30	0.30

Supplementary Table S2. Environmental variables at benthic sampling stations in the Neva River estuary. Each number is the average of three replicates. The raw data were log-transformed (log_{10}) with an added constant of 2. Sal – water salinity (PSU); Temp – water temperature (°C); pH – hydrogen potential; Eh – Oxidation/Reduction Potential (mV); Turb – water turbidity (NTU); Dth – depth (m); SM – concentration of particulate suspended matter above (e) and below (g) thermocline; CHL – chlorophyll *a* concentration in water (mg m^{-3}); PP – plankton primary production (gC m^{-2} day^{-1}); MN – rate of mineralization of organic matter in water column (gC m^{-2} day^{-1}); PPMN – PP to MN ratio.. Community: 1 – biomass of alien polychaetes *Marenzellaria* spp. > 3.84 g wet weight m^{-2}; 2 – biomass of alien polychaetes *Marenzellaria* spp. < 3.84 g wet weight m^{-2}.

Community	Year	Station	Sal	Temp	pH	Eh	Turb	Dth	SMe	SMg	CHL	PP	MN	PPMN
1	2014	2	0.82	0.73	0.97	2.35	0.70	1.18	0.74	0.78	1.20	0.54	0.56	0.46
1	2020	6	0.65	1.05	0.99	2.31	1.03	1.35	0.62	0.75	1.21	0.51	0.56	0.44
1	2017	4	0.64	0.85	0.98	2.37	1.21	1.32	0.74	1.14	1.41	0.66	0.68	0.46
1	2017	6	0.64	0.84	0.99	2.44	0.94	1.36	0.81	0.97	1.32	0.68	0.55	0.57
1	2019	6	0.66	0.76	0.99	2.22	0.80	1.37	0.71	0.73	1.32	0.61	0.61	0.48
1	2018	5	0.68	0.80	0.98	2.42	0.86	1.43	0.71	0.75	1.53	0.53	0.67	0.40
1	2018	6	0.68	0.83	0.98	2.39	0.97	1.37	0.68	0.71	1.35	0.49	0.61	0.40
1	2019	5	0.67	0.71	0.98	2.17	0.70	1.44	0.72	0.68	1.22	0.52	0.55	0.45
1	2016	6	0.66	1.16	1.00	2.45	1.24	1.36	0.81	0.94	1.30	0.52	0.50	0.50
1	2020	5	0.65	0.92	0.98	2.34	0.92	1.42	0.69	0.67	1.47	0.67	0.71	0.46
1	2015	1	0.51	1.29	0.99	2.49	1.41	1.11	0.75	0.75	1.40	0.54	0.55	0.48
1	2014	4	0.82	0.70	0.97	2.32	1.11	1.32	0.66	0.81	1.10	0.46	0.63	0.38
1	2014	6	0.83	0.70	0.97	2.23	1.08	1.36	1.04	1.05	1.28	0.45	0.66	0.37
1	2014	5	0.83	0.68	0.97	2.39	0.87	1.42	0.75	0.85	1.00	0.44	0.64	0.37
1	2017	5	0.64	0.72	0.98	2.44	0.90	1.43	0.73	0.80	1.18	0.58	0.60	0.47
1	2015	6	0.74	1.09	0.98	2.46	1.42	1.37	1.42	1.23	1.22	0.34	0.58	0.33
1	2016	5	0.66	1.13	0.99	2.46	1.24	1.43	0.82	0.91	1.17	0.52	0.41	0.63
1	2015	5	0.75	0.98	0.98	2.45	1.55	1.44	1.04	0.84	1.25	0.47	0.57	0.41
2	2014	7	0.81	0.99	0.97	2.51	0.89	0.94	1.23	1.24	1.27	0.42	0.48	0.42
2	2017	7	0.52	1.25	1.00	2.45	0.92	0.95	0.82	0.82	1.35	0.63	0.49	0.61
2	2019	4	0.65	0.82	0.99	2.33	0.76	1.33	0.70	0.69	1.24	0.50	0.56	0.43
2	2021	1	0.37	1.36	1.00	2.42	1.19	1.08	0.84	0.84	1.47	0.58	0.72	0.41
2	2023	4	0.64	1.31	1.00	2.41	0.99	1.32	0.91	0.91	1.71	0.71	1.00	0.38
2	2022	3	0.37	1.33	0.99	2.42	1.04	1.15	0.83	0.97	1.49	0.60	0.83	0.38
2	2018	7	0.62	1.16	0.99	2.42	0.73	0.98	0.81	0.76	1.47	0.55	0.52	0.50
2	2021	3	0.53	1.34	1.00	2.35	1.40	1.13	0.72	0.72	1.49	0.56	0.68	0.41

Supplementary Table S2. Environmental variables at benthic sampling stations in the Neva River estuary. Each number is the average of three replicates (continued).

Community	Year	Station	Sal	Temp	pH	Eh	Turb	Dth	SMe	SMg	CHL	PP	MN	PPMN
2	2018	1	0.67	0.96	0.98	2.43	0.76	1.11	0.81	0.82	1.83	0.78	0.74	0.50
2	2021	4	0.82	0.99	0.99	1.98	1.55	1.31	0.64	0.87	1.35	0.59	0.60	0.47
2	2022	6	0.79	0.91	0.99	2.41	1.07	1.35	0.85	0.82	1.35	0.56	0.68	0.41
2	2022	4	0.66	1.24	0.98	2.39	1.11	1.31	0.72	0.78	1.35	0.61	0.70	0.43
2	2016	3	0.49	1.32	1.00	2.52	1.09	1.16	0.78	0.78	1.40	0.67	0.53	0.60
2	2023	3	0.44	1.32	0.99	2.01	0.99	1.16	1.08	1.08	1.95	0.85	1.05	0.41
2	2022	2	0.44	1.32	0.99	2.44	1.39	1.20	0.95	0.95	1.44	0.54	0.71	0.39
2	2023	1	0.41	1.32	1.00	2.47	1.25	1.10	0.81	0.81	1.53	0.66	0.80	0.41
2	2018	3	0.67	0.91	0.98	2.42	0.91	1.19	0.71	0.69	1.38	0.65	0.54	0.57
2	2016	7	0.63	1.25	0.99	2.47	1.04	0.94	0.86	0.86	1.50	0.57	0.61	0.45
2	2020	4	0.64	1.13	0.99	1.89	0.94	1.31	0.78	0.94	1.54	0.56	0.78	0.38
2	2021	2	0.66	1.33	0.98	2.38	1.48	1.17	0.80	0.80	1.44	0.60	0.55	0.51
2	2020	3	0.60	1.27	0.98	2.25	1.08	1.15	0.70	0.76	1.41	0.59	0.66	0.44
2	2022	7	0.52	1.32	1.01	2.35	0.81	0.97	0.83	0.83	1.52	0.55	0.67	0.41
2	2020	1	0.58	1.28	0.98	2.35	1.12	1.08	0.67	0.67	1.41	0.56	0.57	0.47
2	2014	3	0.81	0.84	0.97	2.38	0.84	1.15	0.87	0.91	1.08	0.46	0.57	0.40
2	2017	1	0.35	1.32	1.01	2.31	0.61	1.09	0.78	0.78	1.46	0.66	0.60	0.52
2	2017	3	0.43	1.27	0.99	2.34	1.24	1.15	0.74	0.74	1.30	0.57	0.61	0.45
2	2023	5	0.75	1.16	0.98	2.32	1.34	1.42	0.64	0.64	1.38	0.64	0.95	0.37
2	2021	7	0.47	1.35	1.01	2.36	1.33	0.95	0.91	0.91	1.52	0.63	0.56	0.53
2	2023	2	0.46	1.32	1.00	2.39	1.23	1.20	0.95	0.95	1.63	0.67	0.89	0.39
2	2018	2	0.67	0.86	0.98	2.44	0.94	1.22	0.71	0.71	1.30	0.63	0.54	0.55
2	2019	1	0.63	1.10	0.99	2.41	0.91	1.10	0.75	0.73	1.53	0.62	0.45	0.67
2	2016	1	0.38	1.36	1.01	2.54	1.03	1.10	0.83	0.83	1.58	0.64	0.64	0.48
2	2023	7	0.56	1.31	1.00	2.36	0.86	0.95	0.83	0.83	1.58	0.70	0.65	0.51
2	2022	1	0.36	1.34	1.00	2.41	0.88	1.10	0.86	0.86	1.47	0.63	0.71	0.44
2	2019	3	0.64	1.02	0.99	2.21	0.91	1.15	0.78	0.78	1.51	0.64	0.60	0.51
2	2021	5	0.87	0.76	0.99	2.05	0.83	1.41	0.69	0.71	1.25	0.54	0.54	0.48
2	2020	2	0.62	1.25	0.99	2.34	1.29	1.15	0.72	0.72	1.36	0.58	0.76	0.39
2	2016	2	0.65	1.21	0.99	2.55	1.12	1.20	0.70	0.94	1.30	0.54	0.39	0.70

Supplementary Table S2. Environmental variables at benthic sampling stations in the Neva River estuary. Each number is the average of three replicates (continued).

Community	Year	Station	Sal	Temp	pH	Eh	Turb	Dth	SMe	SMg	CHL	PP	MN	PPMN
2	2014	1	0.76	1.06	0.99	2.37	0.90	1.10	0.81	0.88	1.23	0.53	0.63	0.41
2	2017	2	0.59	1.16	0.98	2.45	1.51	1.19	0.79	0.79	1.33	0.56	0.63	0.43
2	2021	6	0.83	0.97	0.99	2.36	0.89	1.35	0.74	0.84	1.35	0.59	0.59	0.47
2	2023	6	0.75	1.20	0.98	2.37	1.12	1.35	0.86	0.86	1.54	0.67	1.04	0.36
2	2019	7	0.59	1.16	0.99	2.36	0.85	0.98	0.83	0.84	1.63	0.59	0.51	0.55
2	2015	3	0.62	1.28	0.98	2.49	1.33	1.17	1.00	1.00	1.24	0.46	0.44	0.51
2	2022	5	0.83	0.69	0.98	2.36	0.80	1.42	0.76	0.76	1.25	0.51	0.85	0.35
2	2018	4	0.68	0.78	0.98	2.19	0.91	1.33	0.68	0.69	1.34	0.61	0.68	0.44
2	2019	2	0.65	0.88	0.99	2.36	0.87	1.22	0.74	0.70	1.38	0.56	0.59	0.46
2	2016	4	0.66	1.16	0.99	2.47	1.03	1.32	0.65	0.87	1.11	0.56	0.46	0.59
2	2015	2	0.63	1.28	0.98	2.37	1.57	1.24	0.79	0.91	1.38	0.56	0.48	0.55
2	2015	7	0.60	1.29	0.99	2.49	1.42	0.99	1.68	1.64	1.28	0.34	0.36	0.41
